# A case report of tinea capitis in infant in first year of life

**DOI:** 10.1186/s12887-019-1433-7

**Published:** 2019-02-22

**Authors:** Narcisa Mandras, Janira Roana, Ornella Cervetti, Michele Panzone, Vivian Tullio

**Affiliations:** 1 Department of Public Health and Pediatrics, University of Turin, Torino, 10126 Italy; 2 Department of Medical Sciences, University Clinic of Dermatology, University of Turin, Torino, Italy

**Keywords:** Pediatric infections, Case report, Tinea capitis, Dermatophytes, *Microsporum canis*, Correct treatment

## Abstract

**Background:**

Tinea capitis is a cutaneous fungal infection common among 3 to 7 year old children but it is rare in the first year of life.

**Case presentation:**

We present a case of a 12-month-old infant with erythematous scalp lesions combined with hair loss. He was suspected of dermatophytosis and mycological analysis of all suspected lesions was performed. Clinical features and culture results confirmed tinea capitis caused by *Microsporum canis.* The infant patient was treated with griseofulvin for 2 months. However, 15 days later at the end of treatment he presented with a single vesicle positive for *M. canis.* Griseofulvin therapy continued for another month. After 3 months of follow-up, no recurrence was observed.

**Conclusions:**

In infant, sometimes tinea capitis is misdiagnosed and underreported because it is similar to other scalp pathologies. Therefore, if erythematous scalp lesions are present, they must be examined from a mycological point of view to inform the differential diagnosis. Once diagnosed, treatment of tinea capitis can pose a dilemma because different factors may influence the choice between equally effective therapies (i.e. safety, age, formulation, cost). This case report suggests that it is important to establish an accurate diagnosis and  treatment for this dermatophytosis to avoid recurrences or therapeutic failures, especially in infants.

**Electronic supplementary material:**

The online version of this article (10.1186/s12887-019-1433-7) contains supplementary material, which is available to authorized users.

## Background

Tinea capitis (TC) is a fungal infection of the scalp and the surrounding skin due to dermatophytes such as *Microsporum* spp. and *Trichophyton* spp. [1, 2]. It is a predominantly dermatophyte infection in children 3 to 7 years old and it is rare in infants in the first year of life [3, 4]. We report a case of an infant with TC admitted to the Medical Science Department, University of Turin (Italy).

### Case presentation

**Fig. 1:**
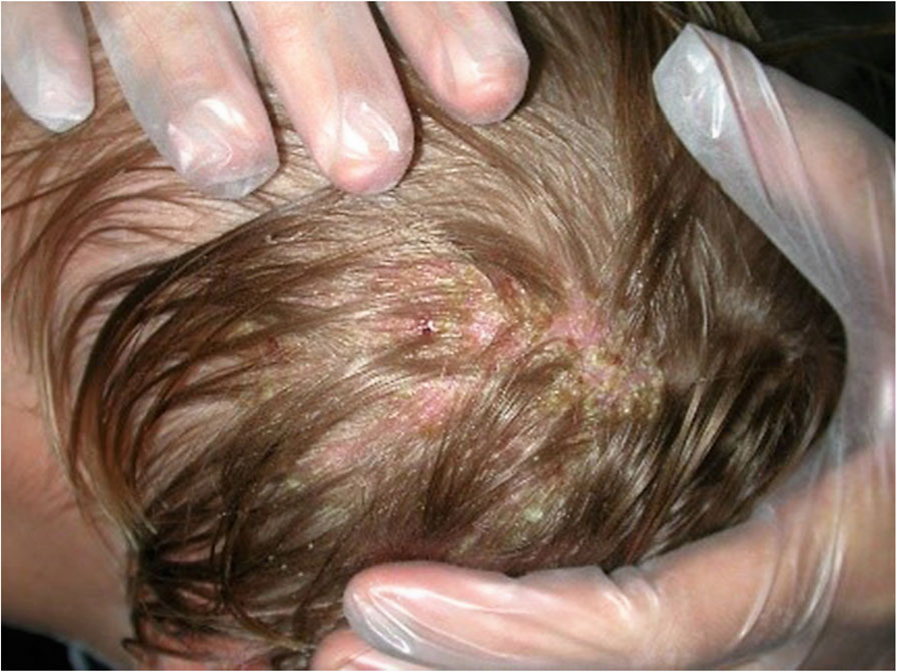
A 12 month-old male infant presented a history of erythematous scalp lesions combined with hair loss

A.M., a 12-month-old male infant, Caucasian, Italian, in good general health, with no history of recent fever or any other symptoms. The patient was evaluated for erythematous scalp lesions and annular patches combined with hair loss (Fig. 1). The infant had not been in contact with animals; her mother and other family members were asymptomatic. No other systemic symptoms were elicited. He was suspected to have a dermatophytosis.

**Fig. 2:**
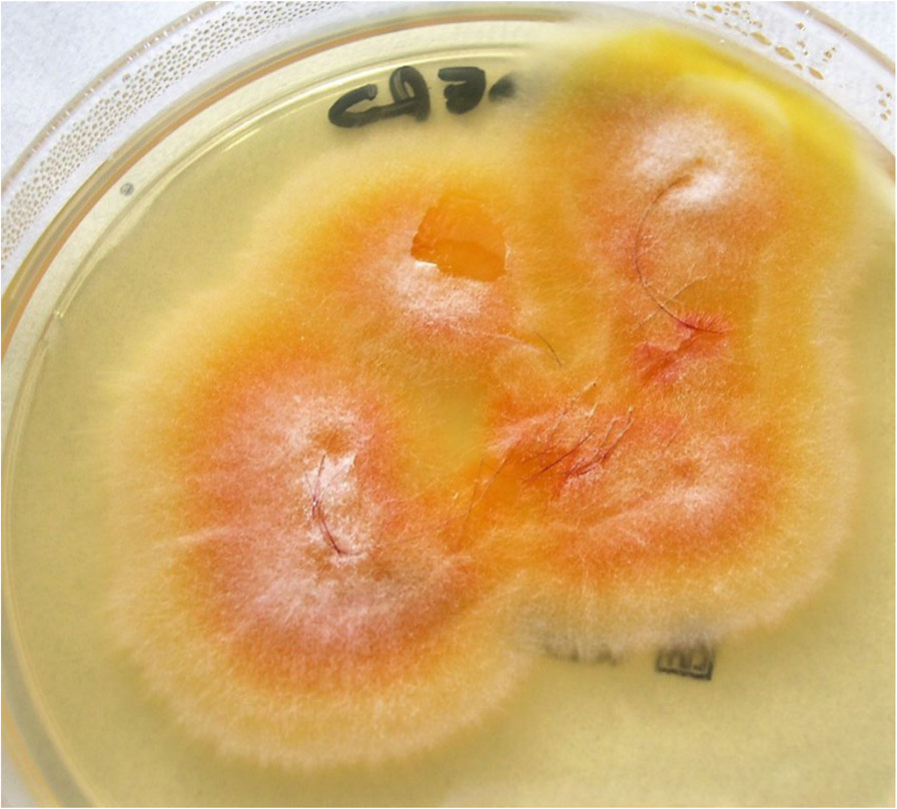
Culture of the specimen revealed the presence of *M. canis* colonies

**Fig. 3:**
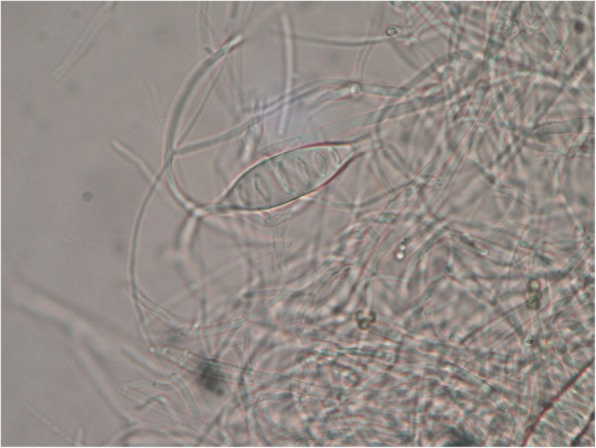
“Spindle” shaped multicellular macroconidia (400x)

The Wood’s light examination revealed a brilliant green fluorescence on the scalp lesions. Mycological analysis of all suspected dermatophyte lesions was performed. Hair and scale samples were collected and examined under a light microscope with 20% *v*/v KOH + 40% v/v DMSO solution in distilled water. A fungal culture was performed into Mycobiotic agar (Merck, KGAA, Germany) to identify dermatophytes. Plates were incubated at 25 °C and examined every 2–3 days for at last 15 days. Mold identification was based on macroscopic and microscopic assessment of colonies [5]. Macroscopic examination revealed some white fluffy spreading colonies (Fig. 2), and a characteristic deep yellow-orange pigment on the reverse. Spindle shaped multicellular macroconidia with thick cell walls were detected on microscopic examination (Fig. 3). Clinical features and culture results reveled TC caused by *Microsporum canis.*

Therapy was started with oral griseofulvin (20 mg/kg/day) with a 2 daily tioconazole cream application. The lesions were also treated with iodized alcohol. At 15-day intervals, the child was subjected to objective examination, including culture tests. The patient was treated for two months in total with both medications and iodized alcohol. After this period of treatment, the first negativization of the culture for *M. canis* was observed. However, 15 days later at the end of treatment, in the area of the lesions, where hair regrowth was observed, the infant patient presented with a single vesicle with growth of *M.canis* on culture. Oral treatment with griseofulvin (20 mg/kg/day) was administered for one month. After this period, all scalp lesions were completely healed and cultures resulted negative for dermatophytes. After 3 months of follow-up, no recurrence was observed (Additional file 1).

## Discussion and conclusions

Epidemiology of TC can be related to geographical location and social, cultural and nutritional factors: in infants, across Europe and the Mediterranean basin, *M. canis* remains the most common organism responsible for TC, with prevalence ranges between 0.23 and 2.6%. *Trichophyton tonsurans* is reported as accounting for 50–90% of dermatophyte scalp isolates in the UK and the USA. In addition, *T. tonsurans* has spread to both South America and West Africa. *T.violaceum* is the most common in Greece and Belgium [6–8].

Tinea capitis in infants likely is more common than is reported and recent literature demonstrates a significant increase. Although the incidence is low, sometimes TC is misdiagnosed and underreported, and differential diagnosis may include seborrheic dermatitis, atopic dermatitis, neonatal lupus, Langerhans cell histiocytosis and syphilis. TC should be suspected in a child with alopecia, pruritus and/or persistent desquamation and thinning hair, and the scalp lesion should be investigated from a mycological point of view [6, 9, 10].

The drug of choice for the treatment of TC is griseofulvin in children. Six to twelve weeks therapy is recommended or until the patient tests negative for fungi (light microscopy and culture). However, the long period of treatment required with this antifungal drug is a significant disadvantage and leads to reduced compliance [8]. Other oral antifungals, specifically fluconazole, itraconazole, ketoconazole, and terbinafine are available and give the advantage of good safety and efficacy profiles, and shorter required duration of treatment of TC caused by *Tricophyton* and *Microsporum*. [8]. Fremerey and Nenoff reported a case of TC in a newborn caused by *T. soudanense* [11]. The patient was initially treated with intravenous fluconazole and topical clotrimazole and octenidine therapy; after one week of treatment, the lesions were resolving [11]. Oral terbinafine, which is often administered in dermatophytosis, is FDA approved only for treatment of TC in children four years of age or older. Oral ketoconazole has been suggested for treatment of dermatophyte infections where griseofulvin is not tolerated. However, Michaels et al. do not consider oral ketoconazole to be as prudent an alternative due to the higher risk of hepatotoxicity [3].

Nevertheless griseofulvin is the cheapest agent in tablet form, has the last potential drug interactions, and is available as a suspension. Among all the alternative drugs to griseofulvin for the treatment of TC, the most promising, as reported by Shemer et al. [12], is fluconazole; in fact, the azole was found in children to have efficacy similar to that griseofulvin in infection due to *M. canis, T. verrucosum* and *T. violaceum*. This drug also reaches high concentrations in the epidermis and persists for several weeks, and is optimal for children because it is supplied as a rectal preparation or syrup and can be used only once weekly [12].

Binder et al. reported that TC caused by *Microsporum* species may be treated with a longer duration of itraconazole and 5 mg/kg/day is used for infantile superficial fungal infections within 3 to 6 weeks [13]. However, griseofulvin remains the drug of choice in infants, because it is efficacious (it can penetrate the hair sheath well), it has lower cost and excellent safety profile and continues to be the better treatment option for TC due to *M. canis* [3, 4, 6, 12].

In the present case report, the patient received oral griseofulvin with a daily wash using tioconazole cream application. The therapy lasted for 3 months with a recurrence, probably indicating a low efficacy of griseofulvin at the doses administered. However, it would be important to understand that there are benefits to the use of other antifungal drugs such as fluconazole and itraconazole in comparison with griseofulvin.

In conclusion, although we have presented a single case of TC by *M. canis* in a 12-month-old infant, this may suggest that it is important to establish an accurate diagnosis and treatment for this dermatophytosis to avoid recurrences or therapeutic failures, especially in infants.

## Additional file

Additional file 1: Timeline. Relevant medical history and interventions, in a 12-month-old male infant, are organized as a timeline. The patient was admitted to the Medical Science Department, University of Turin (Italy) and he was evaluated for erythematous scalp lesions and annular patches combined with hair loss. (DOCX 33 kb)

## Abbreviations

DMSO: Dimethyl sulfoxide
KOH: Potassium hydroxide
TC: Tinea capitis
