# Supplementary material for: A case report of tinea capitis in infant in first year of life
Source: BMC Pediatr. 2019 Feb 22;19:65. doi: 10.1186/s12887-019-1433-7 (PMC6385464; doi:10.1186/s12887-019-1433-7)
Supplement: Supplementary file 1 — Timeline. Relevant medical history and interventions, in a 12-month-old male infant, are organized as a timeline. The patient was admitted to the Medical Science Department, University of Turin (Italy) and he was evaluated for erythematous scalp lesions and annular patches combined with hair loss. (DOCX 33 kb) [file 12887_2019_1433_MOESM1_ESM.docx]

**Timeline for case report**

15 February 2015 Infant patient with erythematous scalp lesions suspected of dermatophytosis. Mycological analysis carried out

14 September Follow-up: no recurrence observed

14 June Scalp lesions healed. Negative cultures

14 May A single vesicle positive for *M.canis* in area of lesions. Oral treatment with griseofulvin (20mg/kg/day)

30 April End of treatment

28 February Diagnosis of tinea capitis by *Microsporum canis*. Treatment (griseofulvin, 20mg/kg/day), 2 daily tioconazole cream application *plus* iodized alcohol.
